# Three new species of the karst-associated snorkel snail genus *Rhiostoma* (Caenogastropoda, Cyclophoridae) from Thailand revealed by integrative taxonomy

**DOI:** 10.3897/zookeys.1296.206215

**Published:** 2026-11-03

**Authors:** Pongpun Prasankok, Santirat Rachawong, Piyatida Pimvichai, Ekgachai Jeratthitikul, Thierry Backeljau

**Affiliations:** 1 School of Biology, Institute of Science, Suranaree University of Technology, Nakhon Ratchasima 30000, Thailand School of Biology, Institute of Science, Suranaree University of Technology Nakhon Ratchasima Thailand; 2 Department of Biology, Faculty of Science, Mahasarakham University, Mahasarakham 44150, Thailand Department of Biology, Faculty of Science, Mahasarakham University Mahasarakham Thailand; 3 Animal Systematics and Molecular Ecology Laboratory, Department of Biology, Faculty of Science, Mahidol University, Bangkok 10400, Thailand Animal Systematics and Molecular Ecology Laboratory, Department of Biology, Faculty of Science, Mahidol University Bangkok Thailand; 4 Royal Belgian Institute of Natural Sciences, Vautierstraat 29, B-1000 Brussels, Belgium Royal Belgian Institute of Natural Sciences Brussels Belgium; 5 Evolutionary Ecology Group, University of Antwerp, Universiteitsplein 1, B-2610 Antwerp, Belgium Evolutionary Ecology Group, University of Antwerp Antwerp Belgium

**Keywords:** Caenogastropoda, DNA barcoding, endemic malacofauna, evolutionary islands, karst habitat, molecular species delimitation, scanning electron microscopy

## Abstract

Integrating morphological and molecular datasets provides a robust framework for validating hidden biodiversity and describing novel taxa. In this study, three new species of the operculate land snail genus *Rhiostoma* are described from the isolated limestone karst ecosystems of northern and northeastern Thailand: *Rhiostoma phuphamanense* Prasankok & Pimvichai, **sp. nov**., *R. erawanense* Prasankok & Pimvichai, **sp. nov**., and *R. lampangense* Prasankok & Pimvichai, **sp. nov**. These novel clades are readily distinguished from all known congeners by discrete combinations of shell morphology (size, shape, and whorl counts). Multi-locus phylogenetic analyses (COI and 16S rRNA) paired with multi-approach species delimitation consistently supported the monophyly and distinct evolutionary clades of these three species, separating them from closely related sister taxa. Genetic divergence data further validated these boundaries, with the minimum uncorrected pairwise p-distance separating the three new species from other *Rhiostoma* clades exceeding 9.4% for the COI gene. Notably, *R. erawanense***sp. nov**. exhibits extreme narrow-range endemism, currently restricted entirely to the microhabitats surrounding Tham Erawan (Erawan cave) in Phrae Province. Ultimately, the discovery of these three highly localized species significantly advances our understanding of Southeast Asian malacofaunal richness and underscores the urgent need for targeted habitat conservation within Thailand’s rapidly degrading regional karst landscapes.

## Introduction

Snorkel snails (Cyclophoridae: *Rhiostoma* Benson, 1860) are operculate land snails native to Indochina and the Malay Peninsula (Benson 1860; Gude 1921). Members of this genus are readily distinguished by a circular aperture, a last whorl with a detached portion that is either absent, short, or moderately extended, a cup-shaped calcareous operculum, and a distinctive backward-bending breathing tube at the sutural ridge near the aperture (Páll-Gergely et al. 2016; Chen et al. 2022). These snails are obligate calciphiles, typically restricted to leaf-litter microhabitats in karst and limestone environments. In Southeast Asia, appro­ximately 31 extant species are recognized, with Thailand a major biodiversity hotspot harboring 21 recorded species (Prasankok 2023; Prasankok et al. 2023; Tongkerd et al. 2023). The remaining ten species occur in neighboring countries, such as Myanmar, Laos, and Vietnam (Haines 1855; Fischer 1891; von Möllendorff 1894; Habe 1965; Solem 1966; Fischer 1973; Do et al. 2020).

Limestone karst ecosystems in Southeast Asia are renowned for their exceptional biodiversity, high levels of micro-endemism, and role as “evolutionary islands” (Schilthuizen et al. 1999; Vermeulen and Whitten 1999; Myers et al. 2000; Schilthuizen 2004; Clements et al. 2008; Liew et al. 2014; Foon et al. 2017; Phung et al. 2018; von Oheimb et al. 2019; Sutcharit et al. 2020; Aulia et al. 2026). In Thailand, limestone karsts are patchily distributed across all geographic regions. In the northern and northeastern regions, extensive and heavily dissected limestone formations have fragmented into isolated karst outliers. These isolated hills exhibit high biodiversity and endemism among cyclophorid snails (Tumpeesuwan and Tumpeesuwan 2015; Nantarat et al. 2019; Páll-Gergely et al. 2021; Tongkerd et al. 2023) and other land snail taxa (Pholyotha et al. 2021; Pholyotha et al. 2023; Boonmachai et al. 2023). Although recent taxonomic efforts in northern Thailand have led to the discovery of five new species (Prasankok 2023; Prasankok et al. 2023; Tongkerd et al. 2023), the malacofauna of this region remains substantially underexplored. To date, only seven *Rhiostoma* species have been documented here: *R. dalyi* Blanford, 1902; *R. ebenozostera* Tongkerd & Panha, 2023; *R. lannaense* Tongkerd & Tumpeesuwan, 2023; *R. panhai* Prasankok, 2023; *R. phunangense* Prasankok & Pimvichai, 2023; *R. platymorpha* Tongkerd & Tumpeesuwan, 2023; and *R. tigrina* Tongkerd & Tumpeesuwan, 2023. Among these, *R. ebenozostera*, *R. lannaense*, and *R. tigrina* are widely distributed, whereas the remaining species exhibit narrow-range endemism restricted to localized karst patches.

Historically, species delimitation within *Rhiostoma* has relied almost exclusively on shell morphology (Kobelt 1902; Habe 1965; Tongkerd et al. 2023). However, shell characteristics such as size, whorl coiling patterns, and coloration can exhibit significant intraspecific variation or, conversely, interspecific convergence. This morphological plasticity in conchological characters in Cyclophoroidea makes accurate species identification challenging, frequently leading to under- or overestimations of true species richness (Lee et al. 2012; Nantarat et al. 2014a, 2014b, 2019; von Oheimb et al. 2019; Cole 2019; Cole et al. 2019; Boonmachai and Nantarat 2020; Hirano et al. 2022; Bhosale et al. 2025). During the past decade, the integration of molecular datasets has become instrumental in resolving cryptic species complexes and refining gastropod taxonomy worldwide. In this study, we conducted an extensive land snail survey focusing on the genus *Rhiostoma* across the northern and northeastern regions of Thailand. We employed an integrative taxonomic approach that combines detailed morphological examination with molecular phylogenetics using two mitochondrial gene fragments (COI and 16S rRNA) to evaluate species boundaries and describe newly discovered taxa. As a result of this integrative approach, we effectively clarify species boundaries and formally describe three new species of *Rhiostoma* to science: *R. phuphama­nense* sp. nov., *R. erawanense* sp. nov., and *R. lampangense* sp. nov.

## Material and methods

### Field sampling and tissue preservation

**Figure 1.**
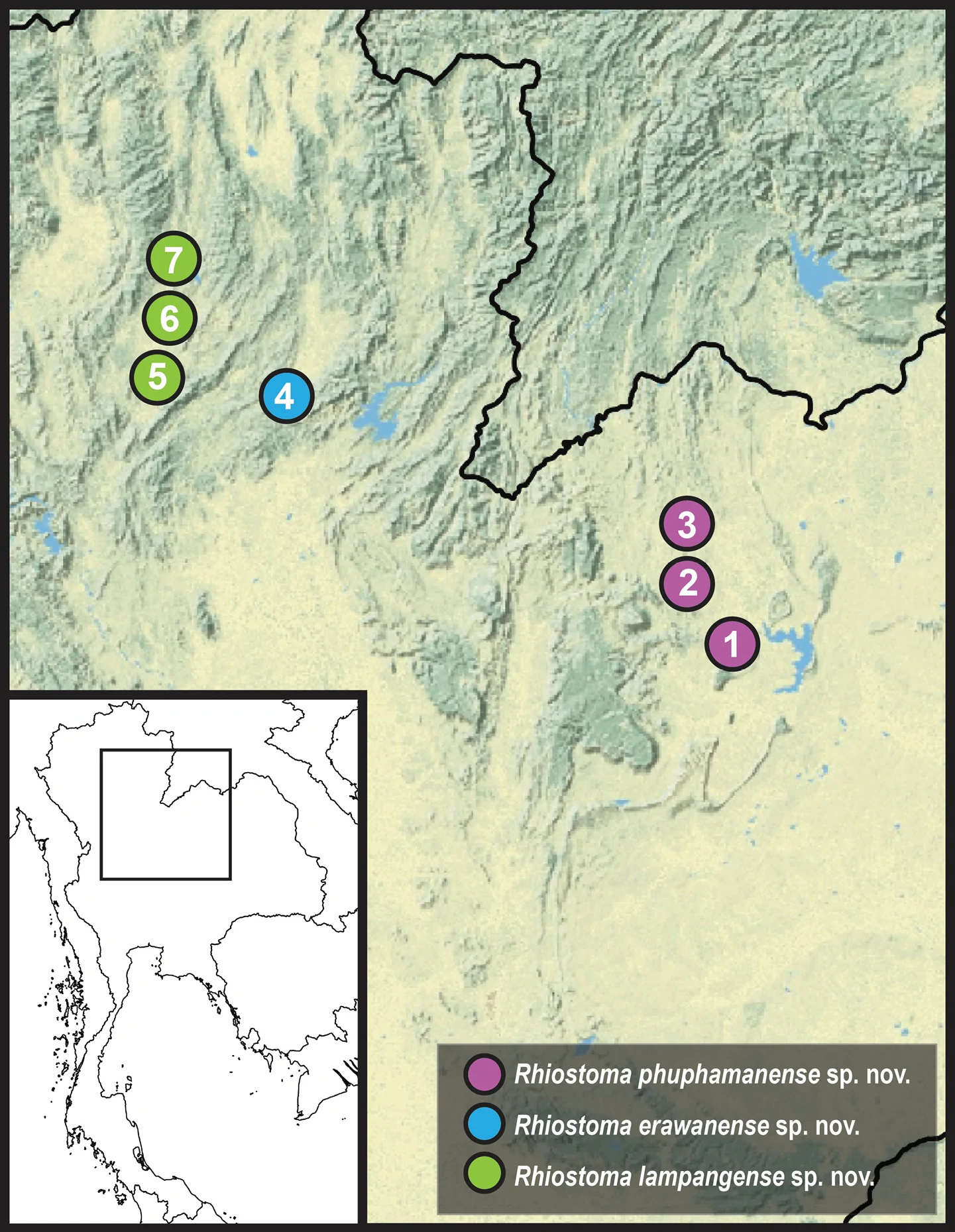
Localities of snorkel snail sampling in this study. 1. Tham Poo Loup, Phuphaman, Khonkaen; 2. Wat Tham Had Nimitre, Loei; 3. Wat Khao San Hin, Loei; 4. Tham Erawan, Phrae; 5. Tham Phra Sabai, Lampang; 6. Tham Itthirit, Lampang; 7. Doi Phrabath, Lampang. Color codes refer to the same clade colors as in Fig. 2.

Field surveys for snorkel snails were conducted across multiple karst landforms throughout northern and northeastern Thailand (Fig. 1) during the wet season. Specimens of *Rhiostoma* were collected using qualitative visual searching across diverse microhabitats, including the soil surface, limestone rock faces, and beneath damp leaf litter. Both living individuals and empty shells were sampled. Initial taxonomic identifications were performed based on shell morphology following Prasankok et al. (2023) and Tongkerd et al. (2023) and verified by direct comparison with type specimens at the Museum of Zoology, Chulalongkorn University (CUMZ), Bangkok, Thailand. For living specimens, soft foot tissues were excised, immediately preserved in 95% (v/v) ethanol, and stored at −20 °C for subsequent molecular analysis. All examined shell vouchers and designated type material were permanently deposited in the CUMZ collection (Table 1). Fieldwork and animal handling protocols were officially approved by the Suranaree University of Technology Animal Care and Use Committee (Approval No. IACUC-67-22).

**Table 1.** List of samples of *Rhiostoma phuphamanense* sp. nov., *R. erawanense* sp. nov., *R. lampangense* sp. nov., and other *Rhiostoma* and *Pterocyclos* species used in this study, with CUMZ voucher numbers and GenBank accession numbers for COI and 16S rRNA. Numbers preceding the localities correspond to the numbered localities shown in the map (Fig. 1).

**Species and localities**	**Coordinates**	**CUMZ numbers**	**Number of specimens**		**Specimen code**	**GenBank accession number**	
			**Morphometric**	**DNA**		**COI**	**16S rRNA**
*Rhiostoma phuphamanense* sp. nov.							
1. Tham Poo Loup, Phuphaman, Khonkaen, Thailand^A^	16°49'45.0"N, 101°59'08.0"E	15477	30	4	MRU51-1	PZ542831	PZ544989
					MRU51-2	PZ542832	PZ544990
					MRU51-3	PZ542833	PZ544991
					MRU51-4	PZ542834	PZ544992
2. Wat Tham Had Nimitre, Loei, Thailand	17°03'21.0"N, 101°54'48.0"E	15478	15	2	MRU43-1	PZ542835	PZ544993
					MRU43-2	PZ542836	PZ544994
3. Wat Khao San Hin, Loei, Thailand	17°10'40.0"N, 101°57'59.0"E	15479	15	2	MRU44-1	PZ542837	PZ544995
					MRU44-2	PZ542838	PZ544996
*R. erawanense* sp. nov.							
4. Tham Erawan, Phrae, Thailand^A^	18°01'59.8"N, 99°30'21.0"E	15480	30	3	MRU24-1	PZ542839	PZ544997
					MRU24-2	PZ542840	PZ544998
					MRU37-1	PZ542841	PZ544999
					MRU37-2	PZ542842	PZ545000
*R. lampangense* sp. nov.							
5. Tham Phra Sabai, Lampang, Thailand^A^	18°05'34.0"N, 99°32'13.0"E	15481	30	4	LP1	PZ542843	PZ545001
					LP3	PZ542844	PZ545002
					LP5	PZ542846	PZ545004
					LP6	PZ542845	PZ545003
6. Tham Itthirit, Lampang, Thailand	18°36'12.3"N, 99°29'37.4"E	15482	15	3	MRU91-2	PZ542847	PZ545005
					MRU91-3	PZ542848	PZ545006
					MRU91-4	PZ542849	PZ545007
7. Doi Phrabath, Lampang, Thailand	18°36'46.4"N, 99°29'51.9"E	15483	15	2	MRU92-1	PZ542850	PZ545008
					MRU92-4	PZ542851	PZ545009
*R. breviocollar* Tongkerd & Tumpeesuwan, 2023							
Khao Tambon, Lopburi, Thailand	15°14'48.9"N, 101°16'40.4"E	14545	30	2	KB10	OP491201*	OP681345**
					KB11	OP491202*	OP681346**
*R. panhai* Prasankok, 2023							
Chao Pho Pratu Pha Shrine, Lampang, Thailand	18°31'0.01"N, 99°49'28.7"E	14387	15	3	MRU35-1	OQ918562***	OQ919148***
					MRU35-2	OQ918563***	OQ919149***
					MRU35-3	OQ918564***	OQ919150***
*R. phunangense* Prasankok & Pimvichai, 2023							
Doi Phunang, Phayao, Thailand	18°51'22.6"N, 100°10'54.9"E	3977	24	2	PN1	OP680760**	OP681317**
					PN3	OP680762**	OP681319**
*R. housei* (Haines, 1855)							
Tham Sriwilai, Saraburi, Thailand	14°42'43.9"N, 100°51'58.6"E	3982	15	1	SL1	OP491242**	OP681326**
Phu Khae, Saraburi, Thailand	14°40'09.0"N, 100°53'12.5"E	14557	15	–	–	–	–
*R. khoratense* Prasankok & Pimvichai, 2023							
Khao Lukchang, Nakhon Ratchasima, Thailand	14°31'33.3"N, 101°21'36.2"E	3981	30	1	LC1	OP680766**	OP681329**
*R. nakwangense* Prasankok & Pimvichai, 2023							
Tham Nakwang, Phetchaburi, Thailand	12°51'25.9"N, 99°56'28.8"E	3988	15	2	NW14	OP680773**	OP681336**
					NW29	OP680776**	OP681339**
*R. abletti* Thach, 2016							
Hot Springs, Meuang Hiam, Houaphanh, Laos	20°5'43.2"N, 103°22'19.6"E	10206	–	1	MRU22	OP491196*	OP681341**
*R. asiphon* Möllendorff, 1893							
Koh Wua Talab, Koh Samui, Surat Thani, Thailand	9°38'06.0"N, 99°40'11.8"E	4767	30	1	MRU16-1	OP491198*	OP681342**
*R. cambodjense* (Morelet, 1875)							
Tham Khao Chakan, Khao Chakan, Sa Kaeo, Thailand	13°39'37.8"N, 102°05'06.7"E	4714	17	1	MRU11-2	OP491204*	OP681348*
*R. cheliopegma* Tongkerd & Tumpeesuwan, 2023							
Tham Takein, Khao Chamao, Rayong, Thailand	12°56'49.3"N, 101°42'34.0"E	3985/2	14	1	TT4	OP491217*	OP681361**
Khao Cha-Ang, Bo Thong, Chonburi, Thailand	13°12'29.8"N, 101°39'6.5"E	4886	11	1	CA1	OP491208*	OP681352**
*R. dalyi* Blanford, 1902							
Hill Near Tham Air Thammachart, Long, Phrae, Thailand	18°17'16.6"N, 100°00'43.8"E	3979	16	2	AT1	OP491219*	OP681363**
					AT9	OP491223*	OP681367**
*R. furfurosum* Tongkerd & Panha, 2023							
Noen Maprang, Phitsanulok, Thailand	16°41'37.9"N, 100°40'44.9"E	3901	30	2	NP3	OP491227*	OP681371**
					NP18	OP491230*	OP681374**
*R. hainesi* Pfeiffer, 1862							
Khao Soi Dao Breeding Centre, Pong Nam Ron, Chanthaburi, Thailand	12°55'19.8"N, 102°14'39.7"E	4457	–	1	SD	OP491235*	OP681379**
Makok Waterfall, Khlung, Chanthaburi, Thailand	12°35'12.0"N, 102°15'21"E	4814	–	1	MRU8-2	OP491234*	OP681378**
*R. haughtoni* Benson, 1860							
Dhammatat Cave, Mawlamyine, Mon, Myanmar	16°30'04.9"N, 97°49'16.6"E	10048	–	1	MRU4	OP491236*	OP681380**
*R. jalorensis* Sykes, 1903							
Tharn Bok Khorani, Ao Luek, Krabi, Thailand	8°23'18.0"N, 98°44'04.0"E	10146	5	1	MRU13-2	OP491245*	OP681382**
Wat Tham Sue, Muang, Krabi, Thailand	8°07'23.4"N, 98°55'21.8"E	10140	8	–	–	–	–
*R. lannaense* Tongkerd & Tumpeesuwan, 2023							
Tham Mae Lana, Pang Mapa, Mae Hong Son, Thailand	19°35'02.3"N, 98°12'48.5"E	10043	5	1	MRU26-1	OP491255*	OP681391**
Wat Pa Tham Wua, Pang Mapa, Mae Hong Son, Thailand	19°31'33.0"N, 98°05'01.3"E	14559	5	–	–	–	–
Tham Pha Chang Mub HinTak, Chiangrai, Thailand	19°54'13.7"N, 100°03'46.8"E	14558	10	–	–	–	–
*R. morleti* Dautzenberg & Fischer, 1906							
Ban Na Wid, Vieng Xai, Houaphanh, Laos	20°26'59.5"N, 104°10'51.2"E	1004/2	–	1	UHL1	OP491258*	OP681393**
*R. platymorpha* Tongkerd & Tumpeesuwan, 2023							
Tham Muang On, Mae On, Chiang Mai, Thailand	18°47'10.6"N, 99°14'17.1"E	4763	30	2	MO47	OP491262*	OP681396**
					MO48	OP491263*	OP681397**
*R. rhothonotaphrosa* Tongkerd & Panha, 2023							
Tham Phet Pho Thong, Klong Hat, Sa Kaeo, Thailand	13°24'49.0"N, 102°19'31.0"E	10172	6	1	MRU17-2	OP491265*	OP681399**
*R. samuiense* Tomlin, 1932							
Tham Wang Thong, Kanom, Nakhon Sri Thamarat, Thailand	9°12'16.0"N, 99°46'26.6"E	4708	6	1	WT	OP491273*	OP681405**
Khao Ma Rong, Bang Sapan, Prachuap Khiri Khan, Thailand	7°47'38.6"N, 99°38'38.5"E	3986	10	–	–	–	–
*R. simplicilabre* Pfeiffer, 1862							
Sapan Waterfall, Bo Kluea, Nan, Thailand	19°11'25.0"N, 101°11'55.0"E	4868	–	2	MRU18-1	OP491274*	OP681406**
					MRU18-2	OP491275*	OP681407**
*R. tigrina* Tongkerd & Tumpeesuwan, 2023							
Wat Tham Pum Tham Pla, Mae Sai, Chiang Rai, Thailand	20°20'53.2"N, 99°51'29.3"E	3909	13	1	MRU34	OP491279*	OP681411**
Tham Saohin Prayanak, Mae Sai, Chiang Rai, Thailand	20°19'24.6"N, 99°51'51.5"E	10193	12	1	MRU31-2	OP491278*	OP681410**
*R. ebenozostera* Tongkerd & Panha, 2023							
Wat Tham Pha Pu, Loei, Thailand	17°34'43.2"N, 101°42'41.2"E	14388	30	3	MRU89-1	PZ542852	PZ545010
					MRU89-2	PZ542853	PZ545011
					MRU89-3	PZ542854	PZ545012
*Pterocyclos blandi* (Benson, 1851)							
Teluic Ewa, Langkawi, Kedah, Malaysia	6°25'02.0"N, 99°45'44"E	4582	–	1	TU	OP491280*	OP681412**
*P. diluvium* Sutcharit & Panha, 2014							
Tam Puttha Kodome, Srinagarindra, Patthalung, Thailand	7°33'36.5"N, 99°53'10.5"E	3812	–	1	PK2	OP491281*	OP681413**
Tham Sumano, Srinagarindra, Patthalung, Thailand	7°35'12.0"N, 99°52'04.0"E	4588	–	1	TS	OP491282*	OP681414**
Gua Cenderawasih, Kangar, Perlis, Malaysia	6°24'45.8"N, 100°11'33.7"E	4592	–	1	GU	OP491283*	OP681415**

* sequences from Tongkerd et al. (2023), ** sequences from Prasankok et al. (2023), *** sequences from Prasankok (2023). ^A^Type localities.

### Morphological and ultrastructural studies

To characterize microstructures, the jaw and radula were isolated from selected specimens. Tissues were cleaned and prepared for scanning electron microscopy (SEM) using a Hitachi S-3400N SEM at the Science and Technology Equipment Center (F11), Suranaree University of Technology.

For shell morphometric analysis, newly collected specimens were evaluated alongside comparative shell-dimension datasets for 18 valid *Rhiostoma* species published by Prasankok (2023) and Prasankok et al. (2023). Each shell was photographed from both apical (top) and apertural (front/side) views using a tripod-mounted Nikon D7200 digital camera under standardized lighting, background, and focal distance conditions. Five linear shell dimensions were quantified using ImageJ ver. 1.53t: shell height (SH), shell width (SW), aperture width (AW), detached-whorl length (DWL), and spire height (SP). DWL was measured exclusively from the apical view, whereas SH, SW, AW, and SP were quantified from the apertural view. To evaluate interspecific morphometric variation among the examined *Rhiostoma* species, a one-way Analysis of Variance (ANOVA) was conducted in IBM SPSS Statistics ver. 25, with statistical significance set at *P* = 0.05.

### DNA extraction, amplification, and sequencing

Total genomic DNA was isolated from preserved foot tissue using the NucleoSpin® Tissue Extraction Kit (Macherey-Nagel Inc.) following the manufacturer’s protocol. Fragments of two mitochondrial genes—cytochrome *c* oxidase subunit I (COI) and 16S ribosomal RNA (16S rRNA)—were amplified. The primer pairs LCO1490/HCO2198 (Folmer et al. 1994) and 16Sar/16Sbr (Kessing et al. 1989) were used for COI and 16S rRNA, respectively. Polymerase chain reaction (PCR) amplification profiles were performed under conditions optimized by Prasankok et al. (2023). Amplicons were verified via agarose gel electrophoresis, purified by gel extraction, and sequenced bi-directionally by Bioneer Inc. (Daejeon, Republic of Korea) on an ABI 3730xl automated genetic analyzer using the initial amplification primers. Raw chromatograms were edited, assembled, and aligned using the MUSCLE algorithm implemented in MEGA ver. 11.0.13 (Tamura et al. 2021). The COI protein-coding sequences were translated into amino acid sequences to verify the absence of stop codons or frameshifts (indicative of pseudogenes). For the 16S rRNA alignment, ambiguous alignment positions and gaps were excluded using the complete deletion option. All novel nucleotide sequences were deposited in GenBank (Accession Nos. COI: PZ542831–PZ542851; 16SrRNA: PZ544989–PZ545009; Table 1).

### Phylogenetic reconstruction

The aligned COI and 16S rRNA sequences were concatenated into a single multi-locus matrix, with each gene treated as a separate data partition. To root the phylogenies, sequences from 23 *Rhiostoma* and two *Pterocyclos* species available on GenBank (Prasankok 2023; Prasankok et al. 2023; Tongkerd et al. 2023) were incorporated, with the *Pterocyclos* species serving as outgroups. Phylogenetic trees were reconstructed using Maximum Likelihood (ML) and Bayesian Inference (BI) criteria. The ML analysis was executed using IQ-TREE ver. 3.1.2 (Wong et al. 2026), employing the built-in ModelFinder (Kalyaanamoorthy et al. 2017) to independently select the best-fit nucleotide substitution models for each partition. Nodal support for the ML tree was evaluated using 1000 ultrafast bootstrap replicates (Hoang et al. 2018). The BI analysis was performed in MrBayes ver. 3.2.7 (Ronquist et al. 2012). For the BI analysis, the GTR+I+G substitution model was applied to the COI partition, and a reversible-jump Markov Chain Monte Carlo (rj-MCMC) approach (nst = mixed) with a gamma-distributed rate variation (rates = gamma) was employed for the 16S rRNA partition. Parameters were completely unlinked across the two partitions to allow independent estimation. The Markov Chain Monte Carlo (MCMC) simulation was run for 10,000,000 generations, with samples collected every 1000 generations. Chain convergence was verified by ensuring the average standard deviation of split frequencies fell well below 0.01. The initial 25% of sampled trees (2500 trees) were discarded as burn-in, and a 50% majority-rule consensus tree was generated to calculate Bayesian posterior probabilities (BPP). Uncorrected pairwise p-distances for the COI gene were calculated in MEGA ver. 11.0.13.

### Species delimitation

To robustly infer putative species boundaries within the genus *Rhiostoma*, three independent molecular species delimitation methods were applied to the COI dataset. The dataset comprised 21 newly generated sequences combined with 27 reference sequences from Prasankok (2023), Prasankok et al. (2023), and Tongkerd et al. (2023), yielding a matrix of 48 COI sequences representing 25 morphospecies. Assemble Species by Automatic Partitioning (ASAP): Distance-based species delimitation was performed on the ASAP web server (https://bioinfo.mnhn.fr/abi/public/asap/) using Jukes-Cantor (JC69), Kimura 2-parameter (K80), and uncorrected pairwise p-distance substitution models. For Poisson Tree Processes (PTP) analysis, tree-based species delimitation was performed using the bPTP web server (https://species.h-its.org/). The non-ultrametric Bayesian Inference (BI) topology generated in MrBayes was used as the input guide tree, and the analysis was run for 100,000 Markov Chain Monte Carlo (MCMC) generations with a 10% burn-in. In the Generalized Mixed Yule Coalescent (GMYC) analysis, for coalescent-based delimitation, a time-calibrated ultrametric input tree was reconstructed using BEAUti ver. 2.7 and implemented in BEAST ver. 2.7 (Bouckaert et al. 2019). A Yule speciation model was selected as the tree prior under a General Time Reversible (GTR) nucleotide substitution model. A relaxed molecular clock with an uncorrelated lognormal distribution was applied to model rate variation among clades. The MCMC simulation was run for 1.0 × 10^8^ generations, with parameters sampled every 1000 generations. After discarding the initial 25% of sampled trees as burn-in, a maximum clade credibility (MCC) tree with median node heights was generated using TreeAnnotator ver. 2.7. The resulting ultrametric MCC tree was analyzed under the single-threshold model on the GMYC web server (https://species.h-its.org/gmyc/).

## Results

### DNA sequence analysis

Sequence variation and dataset characteristics

The aligned COI gene fragment was 658 base pairs (bp) in length, of which 301 sites (45.74%) were variable. No stop codons, insertions, or deletions were detected upon translation, indicating the absence of non-functional nuclear pseudogenes (numts). The aligned 16S rRNA fragment yielded 480 bp, including 178 variable sites (37.08%). The concatenated COI + 16S rRNA dataset yielded a total alignment length of 1138 bp, with 479 variable sites (42.09%).

#### Phylogenetic analysis and divergence

**Figure 2.**
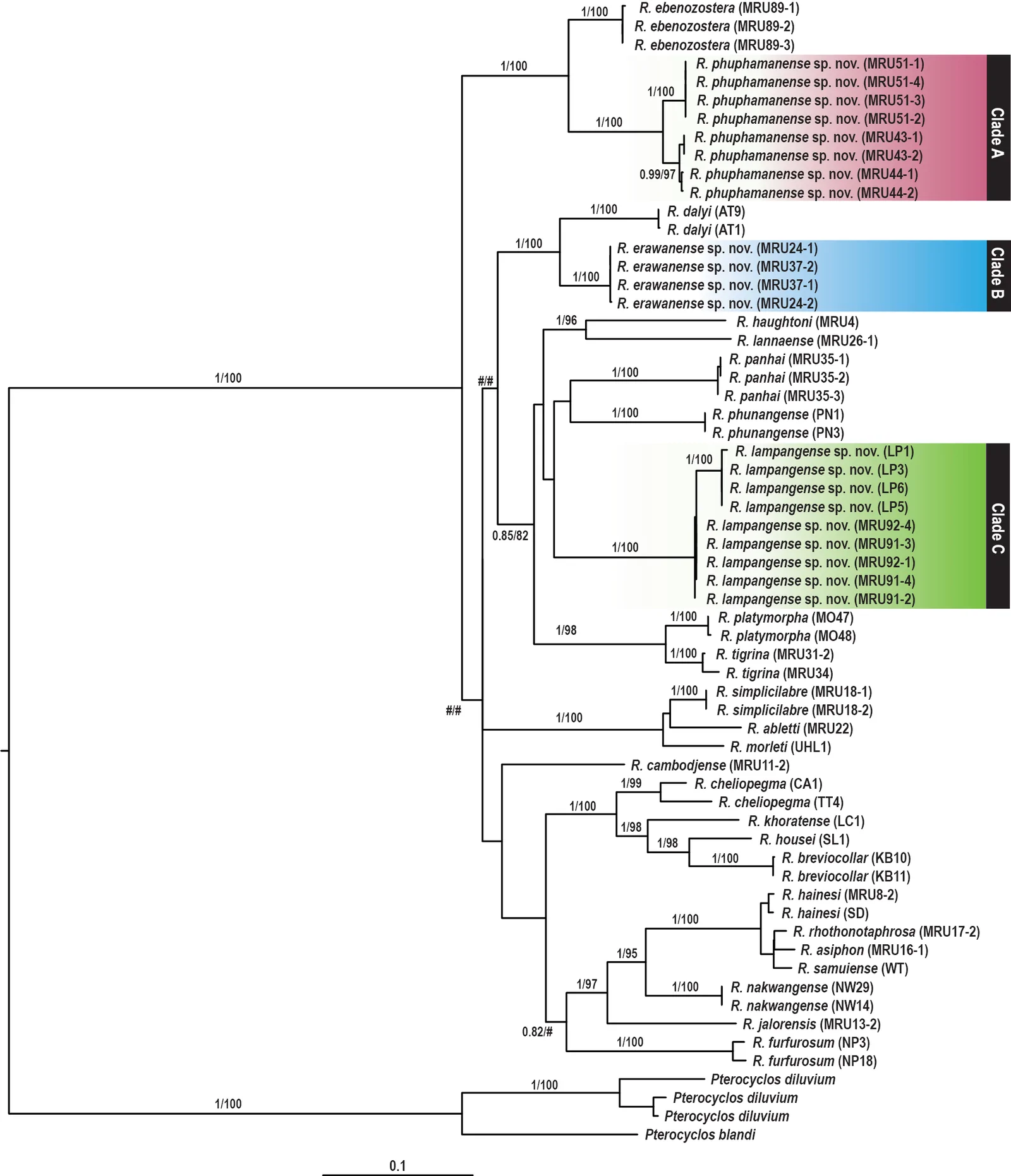
Bayesian Inference (BI) and Maximum likelihood (ML) tree of the genus *Rhiostoma* derived from the combined data of two gene fragments (COI and 16S rRNA). Numbers at nodes indicate branch support based on posterior probabilities (BI)/bootstrapping (ML). Scale bar: 0.1 substitutions per site. Branches with <75% ML bootstrap support and <0.75 posterior probability are indicated with #.

The best-fit nucleotide substitution models selected for the partitioned dataset were TN+F+R3 for COI and TIM2+F+I+G4 for 16S rRNA, which were applied to the ML analysis. For BI tree reconstructions, the GTR+I+G model (COI) and an rj-MCMC approach (16S) were employed. Phylogenetic inferences derived from both ML and BI criteria yielded identical topologies; hence, only the concatenated ML tree is presented with both support values (Fig. 2).

The newly collected specimens clustered into three highly supported, distinct clades (ML BS = 100%; BPP = 1.00), representing three species new to science. *Rhiostoma phuphamanense* sp. nov., comprising individuals from Wat Tham Had Nimitre and Wat Khao San Hin (Loei Province), and Tham Poo Loup (Khonkaen Province), forms a sister taxon to *R. ebenozostera*. *Rhiostoma erawanense* sp. nov., restricted to Tham Erawan (Phrae Province), is recovered as the sister taxon to *R. dalyi*. Furthermore, *R. lampangense* sp. nov., containing specimens from Tham Itthirit, Tham Phra Sabai, and Doi Phrabath (Lampang Province), clusters with *R. panhai* and *R. phunangense*, although this relationship receives weak statistical support (ML BS < 75%; BPP < 0.75).

Interspecific genetic distances calculated from the COI gene fragment revealed a mean uncorrected p-distance of 14.8% (range: 9.4%–20.8%; Table 2) between these three novel clades and previously recognized valid *Rhiostoma* species.

**Table 2.** Uncorrected p-distance of *Rhiostoma phuphamanense* sp. nov., *R. erawanense* sp. nov., *R. lampangense* sp. nov., and related species based on the COI gene. Distances within species are given in bold on the diagonal.

	**1**	**2**	**3**	**4**	**5**	**6**	**7**	**8**	**9**	**10**	**11**	**12**	**13**	**14**	**15**	**16**	**17**	**18**	**19**	**20**	**21**	**22**	**23**	**24**	**25**	**26**
1. *R. phuphamanense* sp. nov.	**2.0**																									
2. *R. erawanense* sp. nov.	13.9	**0**																								
3. *R. lampangense* sp. nov.	16.2	13.8	**1.3**																							
4. *R. ebenozostera*	9.5	10.7	13.1	**0.2**																						
5. *R. dalyi*	15.9	9.4	15.5	14.0	**0**																					
6. *R. haughtoni*	17.4	13.9	16.9	13.1	15.6	**n/c**																				
7. *R. lannaense*	17.4	15.2	16.5	15.3	15.5	14.3	**3.6**																			
8. *R. panhai*	16.1	15.4	13.6	14.7	15.3	16.1	15.7	**0.2**																		
9. *R. phunangense*	16.9	12.4	15.3	13.9	14.8	15.4	14.4	14.4	**0**																	
10. *R. platymorpha*	17.6	15.0	13.6	14.6	15.3	14.7	16.6	14.4	14.7	**0.3**																
11. *R. tigrina*	18.4	14.9	13.7	14.3	16.6	14.5	16.5	14.5	14.8	6.5	**1.8**															
12. *R. simplicilabre*	17.9	14.8	14.4	16.9	14.8	16.4	17.5	16.5	16.2	16.7	17.0	**0**														
13. *R. abletti*	20.8	17.4	17.9	18.8	16.7	18.9	19.2	18.9	18.2	18.0	18.5	8.2	**n/c**													
14. *R. morleti*	19.1	15.8	16.1	16.9	13.6	17.3	16.2	16.7	15.9	17.4	17.3	7.3	9.7	**n/c**												
15. *R. cambodjense*	14.0	11.9	14.5	11.6	14.4	15.3	16.4	14.9	13.6	15.6	16.2	16.8	18.0	15.7	**n/c**											
16. *R. cheliopegma*	16.6	15.6	17.0	14.8	14.4	16.4	17.1	17.8	17.3	17.3	17.2	14.5	16.7	14.8	13.8	**5.3**										
17. *R. khoratense*	16.1	15.7	16.5	15.0	16.4	16.5	17.1	17.1	16.5	17.4	17.8	16.4	17.7	17.5	14.2	11.4	**2.4**									
18. *R. housei*	16.9	15.7	16.9	14.8	15.7	16.5	17.4	16.8	16.8	18.6	17.6	16.9	18.9	16.9	15.3	11.5	9.9	**3.8**								
19. *R. breviocollar*	17.9	15.7	17.1	14.5	15.9	16.9	16.9	18.7	16.6	16.4	17.5	16.3	19.6	16.6	14.9	12.7	12.1	8.9	**0.2**							
20. *R. hainesi*	16.1	16.1	17.7	15.2	17.9	18.3	17.2	17.8	17.4	17.3	17.3	15.8	17.9	15.9	15.9	15.9	15.4	17.7	17.3	**0.7**						
21. *R. rhothonotaphrosa*	16.8	17.4	18.5	15.6	18.4	19.1	17.4	17.9	17.9	17.5	17.4	17.1	19.5	16.8	15.9	17.1	16.5	17.5	17.6	3.3	**1.2**					
22. *R. asiphon*	16.4	16.1	18.5	14.6	18.5	19.0	17.4	18.5	16.9	17.6	17.6	17.4	19.6	17.2	15.8	17.2	15.9	17.9	17.4	3.7	3.6	**1.1**				
23. *R. samuiense*	16.5	17.4	18.1	15.5	17.8	19.2	17.4	17.8	16.9	17.1	17.1	16.7	18.9	15.7	15.8	16.0	15.7	17.8	18.4	3.6	3.3	3.4	**1.8**			
24. *R. nakwangense*	16.0	14.2	15.7	13.9	15.1	16.1	16.5	15.5	15.0	15.0	16.4	16.9	18.5	17.3	13.6	15.1	14.7	15.8	17.0	12.9	13.3	12.8	12.9	**0**		
25. *R. jalorensis*	16.1	15.5	16.3	14.5	16.2	18.3	17.6	15.7	14.7	15.2	16.5	16.8	18.5	17.6	14.2	15.2	14.7	14.8	14.2	13.6	13.9	13.7	13.6	12.9	**5.5**	
26. *R. furfurosum*	18.3	14.8	16.3	15.1	15.7	17.6	17.9	17.1	16.8	16.1	16.6	17.3	18.3	16.9	15.4	13.5	14.0	13.1	14.3	16.7	17.6	17.1	16.7	13.9	14.8	**1.5**

#### Species delimitation

**Figure 3.**
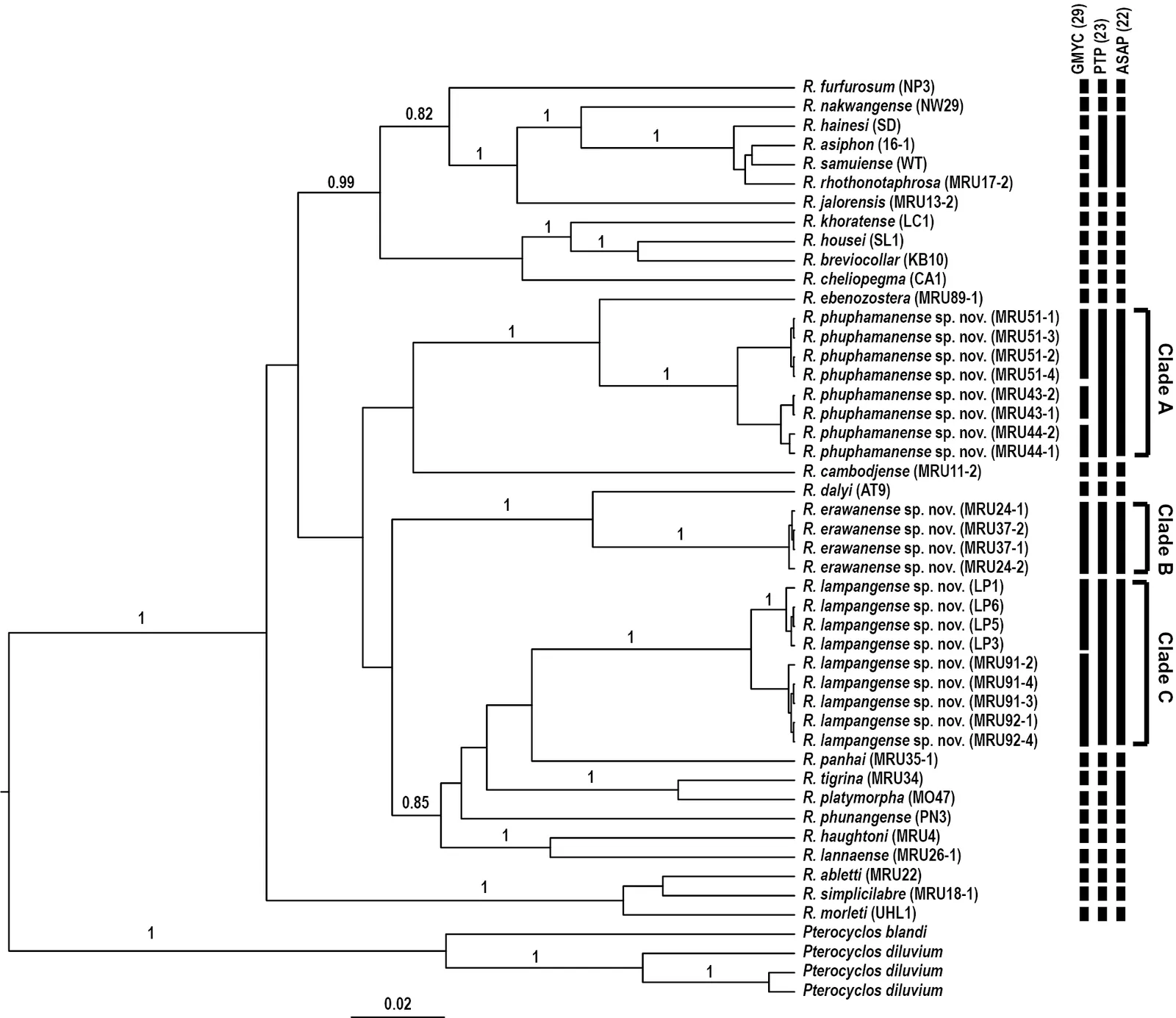
Species delimitation analysis of *Rhiostoma* species based on COI and using GMYC (single threshold), PTP and ASAP. The BI posterior probability support values are provided for each node.

Multi-approach molecular species delimitation yielded varying numbers of operational taxonomic units (OTUs) across the genus *Rhiostoma* (Fig. 3). The single-threshold GMYC model estimated 29 putative OTUs, comprising 6 clusters and 23 singletons (Confidence Interval [CI] = 5–29; log-likelihood of null model = 245.40; log-likelihood of GMYC model = 268.51; Likelihood Ratio Test: *x*^2^ = 46.22, *P* < 0.001). In comparison, the bPTP analysis suggested a slightly more consolidated scheme with 23 putative species. The distance-based ASAP analysis (congruent across uncorrected p-distance, K80, and JC69 models) inferred the most conservative arrangement, yielding 22 putative species at the lowest ASAP score.

Regarding the three novel *Rhiostoma* clades discovered in this study, taxonomic incongruence was observed among the algorithmic approaches. Both the bPTP and ASAP analyses consistently recognized them as three distinct, valid putative species. Conversely, the single-threshold GMYC model highly partitioned these same clades into six distinct molecular OTUs (Fig. 3).

### Shell morphometrics

Univariate statistical analyses demonstrated distinct morphological differentiation among the three newly discovered clades and valid *Rhiostoma* species. The one-way ANOVA revealed highly significant differences (P < 0.05) across all measured shell parameters, including SH, SW, AW, DWL, SP, and the ratio of detached-whorl length-to-aperture width (DWL/AW) (Table 3).

**Table 3.** Comparison of shell morphological characters of closely related *Rhiostoma* species with the new species. Shell morphological characters were tested using one-way ANOVA. Significant differences are marked with * (*P* < 0.05).

**Species**	**Characters (mm)**					
	**Shell Height**	**Shell Width**	**Aperture Width**	**Spire Height**	**Detached-whorl length**	**DWL/AW**
*R. phuphamanense* sp. nov.	13.72–19.10 (16.31 ± 1.3)*	24.14–34.51 (28.96 ± 2.4)*	7.79–11.05 (9.23 ± 0.7)*	4.51–7.22 (5.57 ± 0.5)*	0–7.71 (4.69 ± 1.9)*	0–0.85 (0.514 ± 2.2)*
*R. erawanense* sp. nov.	11.76–16.91 (13.42 ± 1.3)*	19.26–26.62 (21.98 ± 1.8)*	6.18–8.24 (7.08 ± 0.5)*	3.38–6.25 (4.47 ± 0.7)*	4.74–10.33 (7.07 ± 1.4)*	0.69–1.39 (1.00 ± 1.9)*
*R. lampangense* sp. nov.	11.65–16.67 (13.51 ± 1.0)	18.65–25.18 (21.13 ± 1.6)	6.17–8.45 (7.33 ± 0.6)*	4.26–6.52 (5.49 ± 0.5)*	–	–
*R. asiphon*	11.82–16.06 (14.03 ± 1.1)*	20.83–26.74 (24.03 ± 1.6)*	6.74–8.71 (7.76 ± 0.5)*	3.18–4.62 (3.83 ± 0.4)*	0–5.41 (2.58 ± 1.6)*	0–0.65 (0.33 ± 0.2)*
*R. cheliopegma*	10.91–14.47 (12.80 ± 1.0)*	19.85–25.30 (22.12 ± 1.3)*	5.98–7.73 (6.73 ± 0.4)*	3.64–5.00 (4.30 ± 0.4)*	0–4.43 (1.52 ± 1.6)*	0–0.72 (0.24 ± 0.2)*
*R. cambodjense*	12.80–14.62 (13.56 ± 0.6)*	17.73–20.83 (19.29 ± 0.8)*	6.36–7.35 (6.95 ± 0.3)*	4.62–5.91 (5.40 ± 0.4)*	0–3.11 (0.47 ± 0.7)*	0–0.43 (0.06 ± 0.1)*
*R. dalyi*	14.26–20.22 (17.46 ± 1.5)*	23.90–30.74 (28.41 ± 1.9)*	7.65–10.44 (9.06 ± 0.7)*	4.63–7.21 (5.90 ± 0.7)*	6.87–12.01 (9.68 ± 1.6)*	0.81–1.38 (1.07 ± 0.2)*
*R. ebenozostera*	12.79–18.31 (15.01 ± 1.5)*	25.44–30.44 (23.47 ± 1.4)*	6.47–8.46 (7.39 ± 0.5)*	3.97–5.74 (4.84 ± 0.4)*	5.51–10.67 (7.73 ± 1.1)*	0.68–1.32 (1.05 ± 0.2)*
*R. furfurosum*	14.34–18.60 (16.53 ± 1.1)*	23.68–27.57 (28.37 ± 1.3)*	8.31–10.07 (9.07 ± 0.5)*	4.49–6.10 (5.37 ± 0.4)*	4.46–8.88 (7.00 ± 1.2)*	0.49–1.00 (0.77 ± 0.1)*
*R. housei*	13.60–17.94 (15.20 ± 1.3)*	24.78–29.85 (25.45 ± 1.0)*	7.65–9.41 (8.37 ± 0.4)*	3.60–5.59 (4.53 ± 0.5)*	5.78–11.54 (8.24 ± 1.4)*	0.66–1.39 (0.99 ± 0.2)*
*R. jalorensis*	14.63–21.18 (17.20 ± 1.7)*	25.59–34.19 (27.72 ± 1.4)*	6.91–8.82 (8.09 ± 0.5)*	3.46–5.88 (4.46 ± 0.7)*	7.82–12.18 (9.77 ± 1.2)*	1.01–1.47 (1.21 ± 0.2)*
*R. khoratense*	15.22–21.99 (17.68 ± 1.6)*	20.66–29.34 (28.76 ± 2.2)*	8.09–10.74 (9.23 ± 0.7)*	4.26–6.62 (5.45 ± 0.6)*	2.17–9.79 (6.77 ± 1.7)*	0.25–0.99 (0.73 ± 1.8)*
*R. lannaense*	12.28–20.51 (16.34 ± 2.2)*	22.72–30.22 (24.99 ± 2.3)*	6.25–8.01 (7.12 ± 0.6)*	3.38–5.22 (4.24 ± 0.6)*	5.83–14.09 (10.46 ± 2.0)*	0.91–2.01 (1.47 ± 0.3)*
*R. nakwangense*	13.16–19.34 (16.34 ± 1.7)*	20.96–25.74 (26.55 ± 2.2)*	7.06–8.82 (7.93 ± 0.5)*	4.19–5.88 (5.19 ± 0.5)*	5.98–10.49 (8.62 ± 1.3)*	0.75–1.27 (1.09 ± 0.1)*
*R. panhai*	12.06–15.44 (13.75 ± 0.9)*	23.82–32.50 (23.15 ± 1.4)*	6.69–8.01 (7.27 ± 0.4)*	3.53–5.59 (4.64 ± 0.5)*	5.21–8.46 (6.21 ± 1.0)*	0.71–1.24 (0.86 ± 0.2)*
*R. phunangense*	15.00–21.25 (18.31 ± 1.7)*	17.28–22.21 (28.16 ± 2.6)*	7.50–10.81 (9.08 ± 1.0)*	5.22–6.99 (5.99 ± 0.5)*	4.03–10.28 (7.44 ± 1.5)*	0.50–1.08 (0.82 ± 0.1)*
*R. platymorpha*	9.26–12.65 (11.00 ± 0.7)*	28.31–34.93 (19.99 ± 1.0)*	5.66–7.28 (6.52 ± 0.4)*	2.79–5.00 (4.10 ± 0.5)*	1.55–5.64 (3.70 ± 1.0)*	0.22–0.81 (0.57 ± 0.2)*
*R. rhothonotaphrosa*	18.38–22.87 (20.57 ± 1.8)*	22.79–30.96 (32.55 ± 2.3)*	8.24–9.93 (9.36 ± 0.7)*	4.85–6.40 (5.72 ± 0.6)*	12.24–17.60 (15.55 ± 2.1)*	1.49–1.79 (1.66 ± 0.1)*
*R. samuiense*	13.24–21.62 (17.26 ± 2.2)*	20.37–28.53 (26.09 ± 2.1)*	6.62–9.41 (8.03 ± 0.9)*	3.31–6.03 (4.42 ± 0.7)*	6.15–14.31 (10.97 ± 2.3)*	0.75–2.16 (1.39 ± 0.4)*
*R. tigrina*	13.75–21.18 (16.65 ± 2.1)*	25.21–30.83 (25.78 ± 1.8)*	6.03–8.24 (7.50 ± 0.5)*	3.68–5.66 (4.69 ± 0.5)*	5.57–12.46 (8.90 ± 1.5)*	0.70–1.86 (1.19 ± 0.2)*
*R. breviocollar*	12.00–18.44 (15.27 ± 0.10)*	20.10–25.87 (24.41 ± 0.11)*	5.54–7.40 (6.72 ± 0.04)*	4.10–7.25 (5.26 ± 0.06)*	1.42–8.94 (4.49 ± 1.8)*	0.23–1.46 (0.68 ± 0.3)*

### Taxonomy

#### Family Cyclophoridae Gray, 1847

**Subfamily Cyclophorinae Gray, 1847**

**Tribe Pterocyclini Kobelt & Möllendorff, 1897**

##### Genus Rhiostoma

Taxon classificationAnimaliaArchitaenioglossaCyclophoridae

Benson, 1860

C515C793-F285-5B5D-8BDF-193DCB0223AB

###### Synonyms.

For a list of the synonyms and usage of the name, see Tongkerd et al. (2023).

###### Type species.

*Rhiostoma haughtoni* Benson, 1860 by original designation in Benson (1860: 96).

###### Remarks.

*Rhiostoma* is diagnosed by a small to large, heliciform to depressed shell. The detached whorl is absent, or present (short to long, curved, and descending). A prominent breathing device of various forms is present. Peristome double; lip thickened and expanded. Shell color variable, ranging from uniform to zigzag patterns. Operculum calcareous, cup-shaped, anticlockwise multispiral, with an elevated lamella.

##### Rhiostoma phuphamanense

Taxon classificationAnimaliaArchitaenioglossaCyclophoridae

Prasankok & Pimvichai sp. nov.

1784AB26-08B0-56CE-9030-C34FD93104C6

https://zoobank.org/953B2A83-655E-4B67-AA98-E01A67A7A5B3

Figs 4, 7A, B

Rhiostoma ebenozostera Tongkerd et al. 2023 (in part).

###### Material examined.

**Figure 4.**
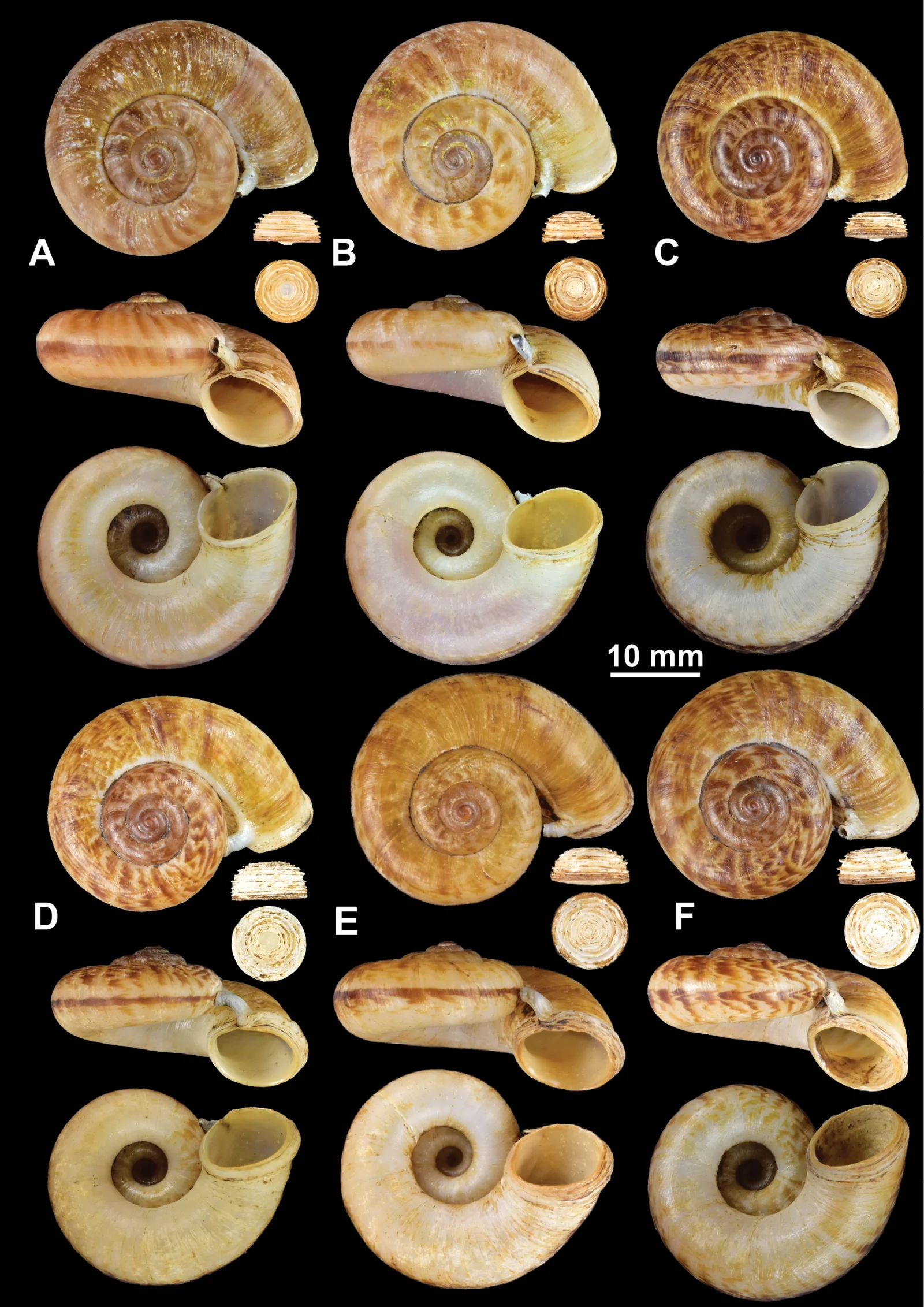
Shell of *Rhiostoma phuphamanense* sp. nov. **A**. Holotype CUMZ 15477 from Tham Poo Loup, Phuphaman, Khonkaen; **B**. Paratype CUMZ 15477 from the type locality; **C, D**. Specimens CUMZ 15479 from Wat Khao San Hin, Loei; **E, F**. Specimens CUMZ 15478 from Wat Tham Had Nimitre, Loei.

***Holotype*** Thailand • 1 shell (height 13.0 mm, width 26.0 mm; Fig. 4A); Khonkaen Province, Chum Phae District, Tham Poo Loup; 16°49'45.0"N, 101°59'08.0"E; P. Prasankok leg.; CUMZ 15477. ***Paratypes*** Thailand • 30 shells; same collection as for holotype; CUMZ 15477 (Fig. 4B).

###### Other material.

Thailand • 15 shells; Loei Province, Wat Khao San Hin; 17°10'40.0"N, 101°57'59.0"E; CUMZ 15479 (Fig. 4C, D) • 15 shells; Loei Province, Wat Tham Had Nimitre; 17°03'21.0"N, 101°54'48.0"E; CUMZ 15478 (Fig. 4E, F).

###### Diagnosis.

The shell is large, thick, and depressed. The detached whorl is short, slightly descending, and shorter than the aperture width (mean detached whorl length / aperture width ratio = 0.51). The breathing device is short, tubular, and incomplete.

###### Differential diagnosis.

*Rhiostoma phuphamanense* sp. nov. differs from the sister taxon, *R. ebenozostera*, by having a larger shell and a shorter detached whorl. In contrast, *R. ebenozostera* features a distinctly longer, descending detached whorl (mean DWL/AW ratio = 1.05).

###### Description.

***Shell***. Shell large (height 11.0–14.0 mm, width 22.5–25.0 mm, detached-whorl length 2.0–5.0 mm), thick, sub-discoidal to discoidal, widely umbilicate. Apex acute; spire little elevated. Whorls 4 to 5 convex, increasing regularly, suture wide and depressed; last whorl rounded; detached whorl very short, slightly descending. Shell surface with thin growth lines. Shell color pale brownish or with thin zigzag streaks, accompanied by a dark brown peripheral band (either thin, thick, or double-thick). Periostracum thick corneous, brownish. Aperture rounded; lip thickened, two layers; inner lip, outer lip, little expanded. Breathing device incomplete tubular, short length, straight; shallow incision. Operculum calcareous, cup-shaped and multispiral.

***Radula***. Taenioglossan radula, each row contains seven teeth with formula 2–1–1–1–2 (Fig. 7A). Central tooth large and semicircular with five cusps, large and flattened at center and smaller cascades laterally. Lateral teeth with four pointed cusps; two inner cusps small but larger than outermost, third cusp triangular and largest, one outer cusp smallest. Marginal teeth tricuspid, two layers; inner marginal teeth located at same level as central tooth, center large and triangular, smaller lateral cusps, the outer marginal teeth located at the edge of the ribbon with a slightly lower position than lateral teeth, located at a lower position than central tooth with central cusp smaller than on inner marginal teeth.

***Jaw***. Jaw sculpture with very strong longitudinal ridges, connected to each other with disordered or ordered parallel slanting transverse ridges, resulting in approximately regular pattern of parallel adjacent lanes of slightly bent oblique rectangles (Fig. 7B). The general structure of the jaw is consistent with the typical morphology observed across the genus *Rhiostoma*.

###### Etymology.

The name comes from the name of the natural park where the type locality of this species is situated.

###### Distribution.

Known from the type locality in Khonkaen Province and the nearby area in Loei Province, Thailand. Specimens were collected under leaf litter. They are sympatric with *Pollicaria* sp. and *Cyclophorus* sp.

###### Remarks.

*Rhiostoma phuphamanense* sp. nov. was previously classified as *R. ebenozostera* (Tongkerd et al. 2023; fig. 29); however, *R. phuphamanense* sp. nov. differs from *R. ebenozostera* by having a larger shell, shorter detached whorl, and shorter incomplete breathing device. This species is supported as clade A in the phylogenetic tree (Fig. 2) and the shell morphological analysis (Table 3).

##### Rhiostoma erawanense

Taxon classificationAnimaliaArchitaenioglossaCyclophoridae

Prasankok & Pimvichai sp. nov.

429616EA-8BDE-56A9-8068-12D8D573C7B1

https://zoobank.org/9C645DE9-5815-4C69-A28B-7287AD847686

Figs 5, 7C, D

Rhiostoma dalyi Tongkerd et al. 2023: fig. 25 (in part).

###### Material examined.

**Figure 5.**
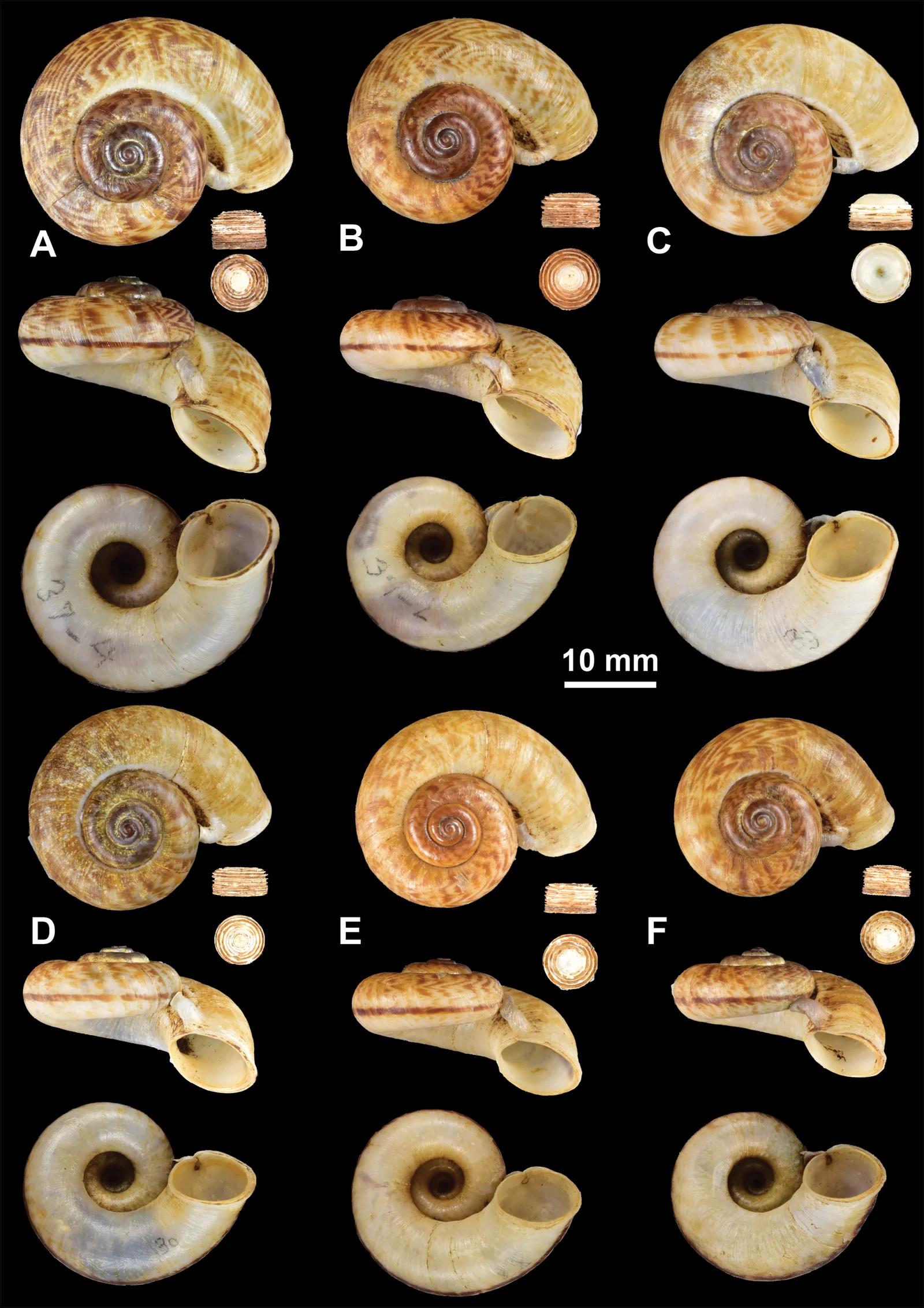
Shell of *Rhiostoma erawanense* sp. nov. **A**. Holotype CUMZ 15480 from Tham Erawan, Phrae; **B–F**. Paratypes CUMZ 15480 from the type locality.

***Holotype*** Thailand • 1 shell (height 10.5 mm, width 21.5 mm; Fig. 5A); Phrae Province, Long District, Tham Erawan; 18°01'59.8"N, 99°30'21.0"E; P. Pimvichai leg.; CUMZ 15480. ***Paratypes*** Thailand • 20 shells; same collection as for Holotype; CUMZ 15480 (Fig. 5B–F).

###### Other material.

Thailand • 10 shells; same data as for holotype; CUMZ 15480/1.

###### Diagnosis.

The shell is medium-sized and covered with a thin, pale brown periostracum. The detached whorl is distinctly separated from the penultimate whorl, curved, descending, and equal to the aperture width (mean detached whorl length / aperture width ratio = 1.00). The breathing device is small, tubular, of medium length, and slightly curved.

###### Differential diagnosis.

*Rhiostoma erawanense* sp. nov. is geographically and phylogenetically close to *R. dalyi* but differs by having a smaller shell, a medium-length, small tubular breathing device, and a detached whorl length equal to the aperture width. In contrast, *R. dalyi* has a larger shell, a longer, curved tubular breathing device, and a mean detached-whorl length-to-aperture width ratio of 1.07.

###### Description.

***Shell***. Shell medium-sized (height 9.0–11.0 mm, width 19.0–21.0 mm, detached-whorl length 4.5–10.0 mm), thick, discoidal, widely umbilicate. Apex acute with dark color; spire little elevated. Whorls 4–5 convex, increasing regularly; suture wide and depressed; last whorl rounded; detached whorl medium-length, curved and descending. Shell surface with thin growth lines. Shell color pale brownish or with thin zigzag streaks, accompanied by a thin, or sometimes thick, dark brown peripheral band. Periostracum thin corneous, brownish. Aperture rounded; lip thickened, two layers; inner lip, outer lip with slightly expanded. Breathing device small, tubular, medium-length and slightly curved; small hole. Operculum calcareous, low cup-shaped and multispiral.

***Radula***. Taenioglossan radula, each row contains seven teeth with formula 2–1–1–1–2 (Fig. 7C). Central tooth large and semicircular with five cusps, large and flattened at center and smaller cascades laterally. Lateral teeth with four pointed cusps; two inner cusps small but larger than outermost, third cusp triangular and largest, one outer cusp smallest. Marginal teeth tricuspid, two layers; inner marginal teeth located at a slightly lower position than central tooth, center large and triangular, smaller lateral cusps, the outer marginal teeth located at the edge of the ribbon with a slightly lower position than lateral teeth, located at a slightly higher position than central tooth with central cusp smaller than on inner marginal teeth.

***Jaw***. Jaw sculpture with very strong longitudinal ridges, connected to each other with disordered or ordered parallel slanting transverse ridges, resulting in approximately regular pattern of parallel adjacent lanes of slightly bent oblique rectangles (Fig. 7D). The general structure of the jaw is consistent with the typical morphology observed across the genus *Rhiostoma*.

###### Etymology.

The name refers to Tham Erawan, the type locality of this species.

###### Distribution.

Known only from the type locality in Phrae Province, Thailand. Specimens were collected under leaf litter near a river.

###### Remarks.

*Rhiostoma erawanense* sp. nov. was previously classified as *R. dalyi* (Tongkerd et al. 2023); however, it is supported as clade B in the phylogenetic tree (Fig. 2) and in the morphological analysis (Table 3).

##### Rhiostoma lampangense

Taxon classificationAnimaliaArchitaenioglossaCyclophoridae

Prasankok & Pimvichai sp. nov.

6A5FBA66-D405-50A9-A55F-D8F77379F8A7

https://zoobank.org/1F869A16-98F2-4AE1-8ED5-E2D83BEA745C

Figs 6, 7E, F

###### Material examined.

**Figure 6.**
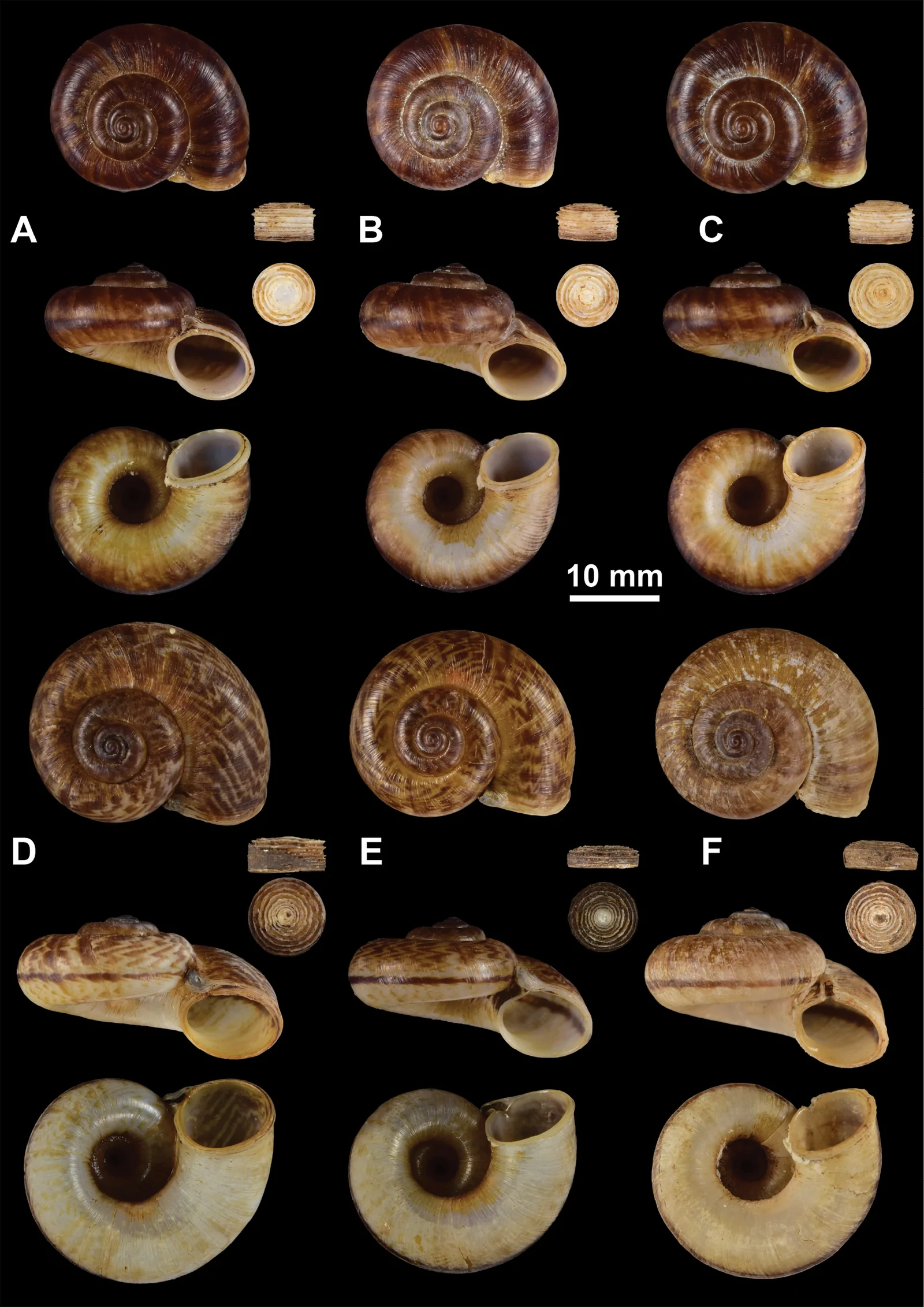
Shell of *Rhiostoma lampangense* sp. nov. **A**. Holotype CUMZ 15481 from Tham Phra Sabai, Lampang; **B, C**. Paratypes CUMZ 15481 from type locality; **D, E**. Specimens CUMZ 15482 from Tham Itthirit, Lampang; **F**. Specimen CUMZ 15483 from Doi Phrabath, Lampang.

***Holotype*** Thailand • 1 shell; (height 11.8 mm, width 20.2 mm; Fig. 6A); Lampang Province, Mae Tha District, Tham Phra Sabai; 18°05'34.0"N, 99°32'13.0"E; P. Pimvichai leg.; CUMZ 15481. ***Paratypes*** Thailand • 20 shells; same collection as for holotype; CUMZ 15481 (Fig. 6B, C).

###### Other material.

Thailand • 10 Shells; same data as for holotype; CUMZ 15481/1 • 15 shells; Lampang Province, Maung Pan District, Ban Khao Subdistrict, Tham Itthirit; 18°36'12.3"N, 99°29'37.4"E; CUMZ 15482 (Fig. 6D, E) • 15 shells; Lampang Province, Maung Pan District, Ban Khao Subdistrict, Doi Phrabath; 18°36'46.4"N, 99°29'51.9"E; CUMZ 15483 (Fig. 6F).

###### Diagnosis.

The shell is small to medium-sized and heliciform, covered with a thick, dark brown periostracum. A detached whorl is completely absent. The breathing device forms a distinct canal that is triangular in shape.

###### Differential diagnosis.

*Rhiostoma lampangense* sp. nov. is geographically close to *R. panhai* but differs in lacking a detached whorl, while *R. panhai* has a medium-length and slightly descending detached whorl (mean detached-whorl length: aperture width ratio = 0.86).

###### Description.

***Shell***. Shell small to medium-sized (height 14.1–17.5 mm, width 23.4–27.0 mm), thick, heliciform, widely umbilicate. Apex acute; spire elevated. Whorl 4–5 convex, increasing regularly; suture wide and depressed; last whorl rounded, without detached whorl. Shell surface with thin growth lines. Shell color: highly variable among populations. Type locality: dark brownish with a dark brown peripheral band (usually thick to double-thick, but sometimes thin). Tham Itthirit: pale brownish or with zigzag streaks, accompanied by a dark brown peripheral band (thin or sometimes thick). Doi Phrabath: pale brown with thin zigzag streaks, and a dark brown peripheral band (thin, thick, or double-thick). Aperture rounded; lip thickened, two layers; inner lip; with or without shallow incision, outer lip; breathing device short, wide, triangular canal located at base near suture. Operculum calcareous, low cup-shaped, and anticlockwise multispiral.

**Figure 7.**
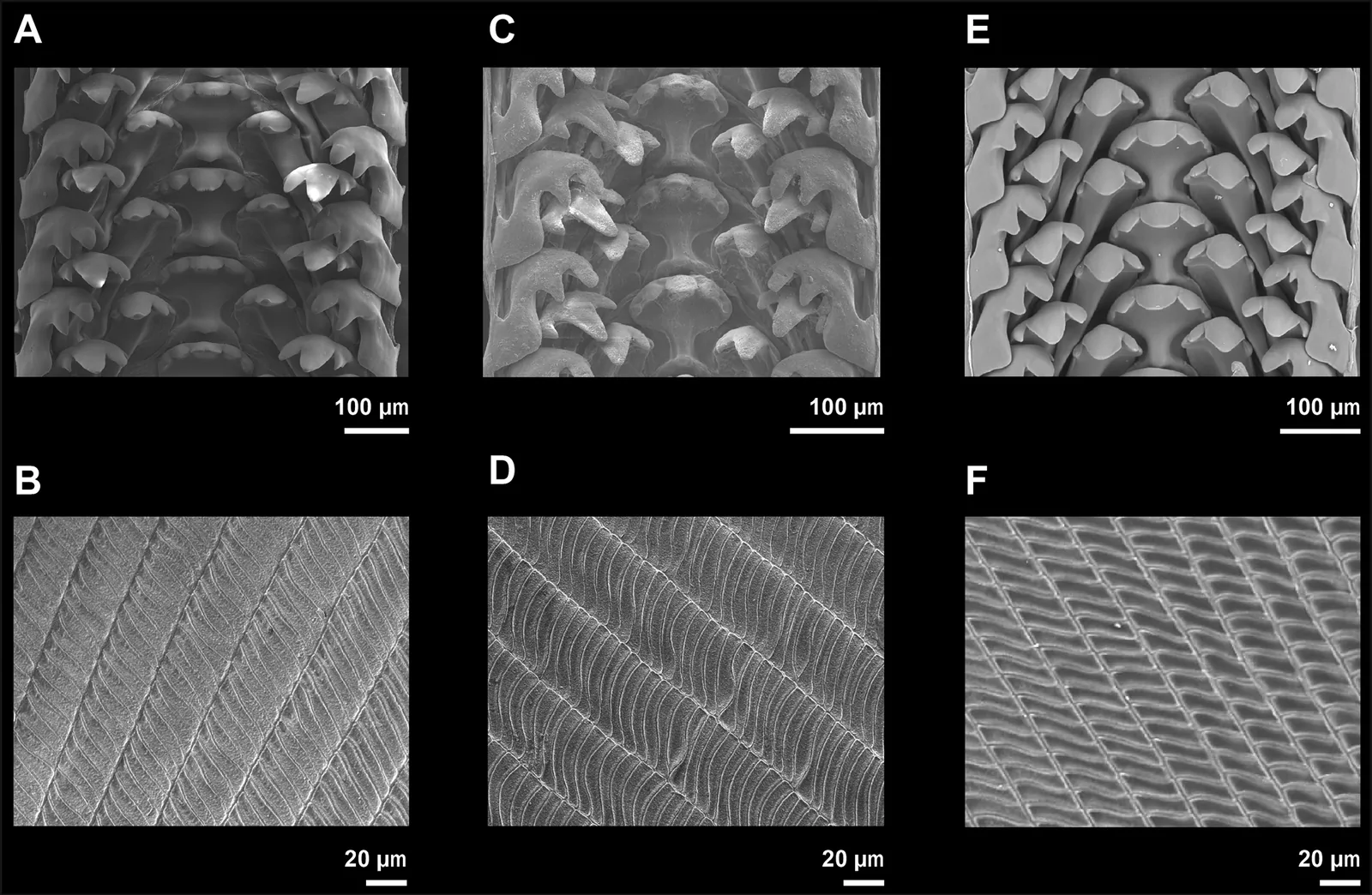
Scanning electron micrographs of radula (**A, C, E**) and microsculpture of the jaw (**B, D, F**) of three new species of *Rhiostoma*. **A, B**. *R. phuphamanense* sp. nov. from Tham Poo Loup, Phuphaman, Khonkaen (clade A); **C, D**. *R. erawanense* sp. nov. from Tham Erawan, Phrae (clade B); **E, F**. *R. lampangense* sp. nov. from Tham Phra Sabai, Lampang (clade C).

***Radula***. Taenioglossan radula, each row contains seven teeth with formula 2–1–1–1–2 (Fig. 7D). Central tooth large and semicircular with five cusps, large and flattened at center and smaller cascades laterally. Lateral teeth with four pointed cusps; two inner cusps small but larger than outermost, third cusp triangular and largest, one outer cusp smallest. Marginal teeth tricuspid, two layers; inner marginal teeth l located at a slightly lower position than central tooth, center large and triangular, smaller lateral cusps, the outer marginal teeth located at the edge of the ribbon with a slightly lower position than lateral teeth, located at same level as central tooth with central cusp smaller than on inner marginal teeth.

***Jaw***. Jaw sculpture with very strong longitudinal ridges, connected to each other with regular parallel slanting transverse ridges, resulting in approximately regular pattern of parallel adjacent lanes of slightly bent oblique rectangles (Fig. 7F). The general structure of the jaw is similar to that of *R. erawanense* sp. nov. and *R. phuphamanense* sp. nov. but differs from *R. lampangense* sp. nov. by having shorter, parallel, slanting transverse ridges.

###### Etymology.

The name refers to Lampang Province, where the type locality of this species is located.

###### Distribution.

Lampang Province, Thailand. Specimens were collected under leaf litter near Limestone Mountain.

###### Remarks.

*Rhiostoma lampangense* sp. nov. clusters with *R. panhai* and *R. phunangense* but is distinguished by its complete lack of a detached whorl, a small- to medium-sized heliciform shell with a dark brown periostracum, and a triangular breathing canal. In contrast, *R. panhai* has a medium-sized shell with a short-to-medium detached whorl (mean detached whorl length/aperture width ratio = 0.86), a dark brown shell with paler zigzag streaks, and a small, slender, curved tubular breathing device (Prasankok 2023). Similarly, *R. phunangense* differs from *R. lampangense* sp. nov. by having a medium-to-large shell with a medium-length detached whorl (mean detached whorl length/aperture width ratio = 0.82), a dark brown shell with paler zigzag streaks, and a larger, curved, medium-length tubular breathing device (Prasankok et al. 2023). This species is supported as clade C in the phylogenetic tree (Fig. 2) and in the shell morphological analysis (Table 3).

## Discussion

### Taxonomic implications and diversity of Thai Pterocyclini

Integrative phylogenetic and morphological analyses executed in this study successfully validated the discovery of three novel *Rhiostoma* species in Thailand. Two clades from the northern region are formally recognized as *R. lampan­gense* sp. nov. and *R. erawanense* sp. nov., while the third clade from the northeastern region is described as *R. phuphamanense* sp. nov. This discovery significantly expands the known species richness of *Rhiostoma*, positioning it as one of the most radiation-dense genera within the tribe Pterocyclini. Globally, Pterocyclini diversity is predominantly driven by *Pterocyclos* (~51 species), *Rhiostoma* (now 34 species), and *Spiraculum* (~27 species) (Kobelt 1902; Thiele 1929; Wenz 1938; Egorov 2009; Prasankok 2023; Prasankok et al. 2023; Tongkerd et al. 2023).

Although the single-threshold GMYC model delineated six potential species-level entities, we conservatively recognize only three distinct new species (*R. phuphamanense* sp. nov., *R. erawanense* sp. nov., and *R. lampangense* sp. nov.), a taxonomic decision that is entirely congruent with the results from both ASAP and bPTP analyses. Tree-based, single-locus delimitation methods such as GMYC are well documented to over-split clades by confounding population-level structure or phylogeographical patterns with true speciation events (Talavera et al. 2013).

Our findings emphasize the exceptional biodiversity and high degree of localized micro-endemism characterizing Thailand’s land-operculate malacofauna, particularly within isolated limestone karst networks. Currently, northern and northeastern Thailand shelter ten documented *Rhiostoma* species, several of which exhibit strict narrow-range endemism restricted to single karst towers or isolated mountain patches (Prasankok 2023; Prasankok et al. 2023; Tongkerd et al. 2023).

### Conservation threats to karst endemic malacofauna

The hyper-localized endemism observed in these obligate calciphilic snails renders them exceptionally vulnerable to anthropogenic disturbances. Tropical limestone outcrops function as terrestrial “evolutionary islands,” promoting rapid speciation but offering zero resilience against habitat fragmentation (Schilthuizen et al. 2003; Clements et al. 2006; Foon et al. 2017; bin Marzuki et al. 2021; Boonmachai et al. 2023). In Southeast Asia, accelerating agricultural expansion, quarrying, and wildfire encroachments significantly degrade these vulnerable ecosystems, driving undocumented endemic clades to the brink of extinction (Schilthuizen et al. 2005; Sodhi et al. 2010; Tripathy et al. 2021; Boonmachai et al. 2024). Consequently, comprehensive biodiversity inventories, such as the baseline data established in this study, are vital for designing site-specific conservation frameworks to preserve these highly threatened, non-pulmonate terrestrial gastropods.

### Morphological and ultrastructural differentiation

Standard morphometric variables, specifically SH, SW, AW, DWL, SP, and the diagnostic DWL/AW ratio, proved highly reliable for distinguishing the three new taxa from all existing congeners. The utility of the DWL/AW ratio as a primary diagnostic criterion aligns precisely with patterns documented in recent *Rhiostoma* revisions (Prasankok et al. 2023; Tongkerd et al. 2023). Although SEM examinations of the radula and jaw microsculpture showed subtle structural variations, the overall structures of both the jaw and radula remain consistent with the typical morphology observed across the genus *Rhiostoma*.

### Phylogenetic relationships and genetic divergence

Multi-locus molecular phylogenies yielded robust topological support for the evolutionary placement of the new species within *Rhiostoma*. *Rhiostoma phuphamanense* sp. nov. was recovered with high nodal support as the sister group to *R. ebenozostera*. Similarly, *R. erawanense* sp. nov. formed a well-supported sister-group relationship with *R. dalyi*. Despite their close phylogenetic affinities, both new species exhibit distinct morphological breaks from their respective sister taxa, explicitly defined by a substantially shorter detached whorl and a lower DWL/AW ratio. Meanwhile, *R. lampangense* sp. nov. clustered with *R. panhai* and *R. phunangense*, yet it is easily differentiated by its completely appressed last whorl (exhibiting a total absence of a detached whorl) and a distinctly small, dark-to-melanistic shell. Concordantly, uncorrected COI pairwise p-distances separating these three new species from their closest relatives substantially exceeded the established genus-wide interspecific mean for *Rhiostoma*. Taken together, the complete congruence between discrete macromorphological characters (particularly shell and detached whorl morphology) and deep mitochondrial divergence unequivocally confirms the evolutionary validity of these three new species.

## Supplementary Material

XML Treatment for Rhiostoma

XML Treatment for Rhiostoma phuphamanense

XML Treatment for Rhiostoma erawanense

XML Treatment for Rhiostoma lampangense

## Additional information

### Artificial Intelligence (AI) use

The authors accept full responsibility for the content of the manuscript, including the disclosure of any use of AI.

No AI tools were used in the preparation of this manuscript.

### Author ORCIDs

P. Prasankok https://orcid.org/0000-0001-5967-223X

S. Rachawong https://orcid.org/0009-0004-0107-2089

P. Pimvichai https://orcid.org/0000-0001-9765-821X

E. Jeratthitikul https://orcid.org/0000-0002-3477-9548

T. Backeljau https://orcid.org/0000-0002-9057-9727

### Data availability

The data that support this study are available in the article.
